# Precipitation of a new platelet phase during the quenching of an Al-Zn-Mg-Cu alloy

**DOI:** 10.1038/srep23109

**Published:** 2016-03-16

**Authors:** Yong Zhang, Matthew Weyland, Benjamin Milkereit, Michael Reich, Paul A. Rometsch

**Affiliations:** 1 Department of Materials Science and Engineering, Monash University, Clayton, VIC 3800, Australia; 2 Monash Centre for Electron Microscopy, Monash University, Clayton, VIC 3800, Australia; 3 Chair of Materials Science, Faculty of Marine Technology and Mechanical Engineering, University of Rostock, 18051 Rostock, Germany; 4 CALOR – Research Competence Center Calorimetry and Thermal Analysis Rostock, Faculty for Interdisciplinary Research, Department Light, Life and Matter, University of Rostock, 18051 Rostock, Germany

## Abstract

A previously undescribed high aspect ratio strengthening platelet phase, herein named the Y-phase, has been identified in a commercial Al-Zn-Mg-Cu alloy. Differential scanning calorimetry indicates that this phase only precipitates at temperature and cooling rate of about 150–250 °C and 0.05–300 K/s, respectively. This precipitate is shown to be responsible for a noticeable improvement in mechanical properties. Aberration corrected scanning transmission electron microscopy demonstrates the minimal thickness (~1.4 nm) precipitate plates are isostructural to those of the T_1_ (Al_2_CuLi) phase observed in Al-Cu-Li alloys. Low voltage chemical analysis by energy dispersive X-ray spectroscopy and electron energy loss spectroscopy gives evidence of the spatial partitioning of the Al, Cu and Zn within the Y-phase, as well as demonstrating the incorporation of a small amount of Mg.

The quench sensitivity of 7xxx alloys has been extensively studied under various cooling conditions in an effort to understand the evolution of the structure-property relationship. Fink and Willey showed in the 1940s that the corrosion resistance behaviour of alloy AA7075 changes with different cooling conditions^1^. Hatch demonstrated that, in the temperature range of 290–400 °C, the tensile strengths of different aluminium alloys changes vastly with average cooling rate due to the promotion of the growth of different phases^2^. Relatively few papers have been published focusing on the nature of the quench-induced phases in 7xxx alloys. Deschamps and Bréchet demonstrated that the equilibrium M-phase nucleates on Cr- or Zr- containing dispersoids in alloy AA7010 during air-cooling^3^. In 2009, Deschamps *et al.* investigated the influence of cooling rate on precipitation in a medium strength 7xxx alloy without Cu (AA7020). They found that the quench-induced precipitation occurs in the temperature range between 320 to 220 °C at cooling rates ranging from 0.1 to 3 K/s^4^. Tang *et al.* proposed that the T and S phases precipitate on grain/subgrain boundaries in a 7xxx alloy during slow cooling^5^.

Recent improvements in controlled heat treatment techniques and experimental design have enabled increasingly comprehensive exploration of temperature, cooling and precipitate -growth in practical timescales. A novel differential scanning calorimetry (DSC) method was developed by Milkereit *et al.* and used to construct novel continuous cooling precipitation (CCP) diagrams for a range of 6xxx and 7xxx series alloys, including AA7020 and AA7150^6^,^7^,^8^. Following this, Zhang *et al.* demonstrated that in the Al-Zn-Mg-Cu alloy AA7150 there are at least three types of quench-induced phases in different temperature ranges over a wide range of continuous cooling rates^8^. This study identified a high temperature reaction at a cooling rate range of about 0.005 to 2 K/s from about 450 °C down to 350 °C, which corresponds to the growth of S-phase (Al_2_CuMg). A medium temperature reaction, corresponding to the η-phase (based on MgZn_2_, but with potential variations), reaches its maximum intensity at about 0.25 K/s and occurrs from about 375 °C down to 250 °C. Finally, a low temperature reaction was detected in the temperature range from ~250 °C down to 150 °C at cooling rates above 0.03 K/s. However, the nature of this phase was not unambiguously verified, due to its small volume fraction and size. The purpose of the current work is to identify this low temperature reaction phase using a combination of DSC, conventional transmission electron microscopy (TEM), and aberration corrected and analytical scanning transmission electron microscopy (STEM) techniques. It also seeks to quantify the effect of this phase on the mechanical properties.

## Materials and Methods

Samples were cut from the centre layer of a commercially produced 80 mm thick AA7150 alloy plate (Al-0.02Si-0.05Fe-2.04Cu-2.15Mg-6.33Zn-0.12Zr in wt%), supplied by the Aluminum Corporation of China Ltd (Chalco).

Samples were solutionised at 460 °C for 1 hour and then slowly heated up to 480 °C within a differential scanning calorimeter (DSC) device and held at this temperature for another hour. Immediately after the solution treatment, the materials were continuously cooled in the same specialised DSC over a range of cooling rates from 0.005 to 3 K/s. A quenching dilatometer was used to achieve cooling rates above 3 K/s. Details of the specialised DSC devices are given elsewhere^7^,^8^.

Tensile testing was carried out in a quenching and deformation dilatometer (type Bähr DIL 805 A/D) at a strain rate of 0.1 s^−1^ immediately after quenching and/or ageing (see supplementary materials for exact geometry). An Instron 8502 servo hydraulic tensile testing machine was used to test the strength of the rapidly quenched samples (≥300 K/s). The tensile test samples were cut from the centre layer of the plate and all tested in the same direction. After solution annealing, the tensile test samples were cooled linearly using the quenching dilatometer. The only exceptions were the fastest two cooling rates, which were carried out by quenching in water at 50 °C and 25 °C, as these rates were not achievable in the dilatometer. As such, for these two conditions, the cooling was not strictly linear. However, based on literature^2^, for a sample thickness of 2 mm and water temperatures of 50 °C and 25 °C, cooling rates are estimated to be about 300 and 1000 K/s respectively. As-quenched tensile strengths and T6 aged strengths (after immediate artificial ageing at 120 °C for 24 hours) were both determined with three samples per condition. Microstructural characterisation was carried out on samples continuously cooled at 10 and 30 K/s. The constructed CCP diagram^8^ (see supplementary materials) for the same alloy indicates that, these cooling rate conditions should result in near-maximum amounts of low temperature phase precipitation while being sufficiently fast to avoid the high temperature reaction completely and minimise the medium temperature reaction^8^.

Conventional bright field (BF) TEM was carried out on an FEI Tecnai T20, operating at 200 kV. High angle annular dark field scanning transmission electron microscopy (HAADF-STEM), electron energy loss spectroscopy (EELS) and energy dispersive X-ray (EDX) spectrum imaging (SI) were carried out on a dual aberration corrected (STEM and TEM) FEI Titan^3^ 80–300 kV microscope. For high resolution HAADF imaging the instrument was operated at 300 kV with a convergence semi-angle of 15 mrad, leading to a diffraction limited (Gaussian) probe diameter of ~0.12 nm. In order to minimise beam damage, analytical spectrum images were acquired at a reduced accelerating voltage of 80 kV, where a convergence semi-angle of 18 mrad was used leading to a diffraction limited probe size of ~0.23 nm. Images were collected on a Fischione HAADF detector with a collection angle of 57–220 mrad. Specimen thickness, and suitability for EELS, was determined by matching simulation to position averaged convergent beam electron diffraction (PACBED). All HRSTEM images are presented in unfiltered form. The EELS data were collected using a Tridiem Gatan Imaging Filter (GIF), with a collection semi-angle of 77 mrad. The EDX spectra were acquired using a Bruker Quantax 400 windowless 60 mm^2^ SDD detector. The EELS and EDX spectra were processed via Gatan Digital Micrograph (DM) and custom routines coded in IDL. Spatial drift was compensated after acquisition by using cross correlation in the plate direction, and plate/matrix average spectra were generated using digital masks generated from simultaneously acquired STEM images.

## Results and Discussion

**Figure 1:**
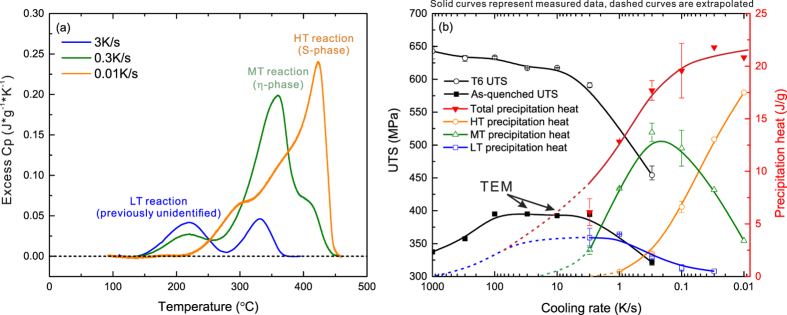
(**a**) Excess specific heat capacity curves for specimens cooled at 3, 0.3 and 0.01 K/s. (**b**) Precipitation heats for total precipitation, the high temperature (HT) S-phase, the medium temperature (MT) η-phase and the low temperature (LT) reaction during continuous cooling covering five orders of magnitude in cooling rate. Ultimate tensile strength (UTS) values are shown for as-quenched and T6 conditions.

Figure 1(a) demonstrates the DSC detected excess specific heat for specimens cooled at three selected cooling rates. Three major exothermal precipitation peaks cover different temperature ranges, with the precipitation behaviour dominated by different peaks at different cooling rates. At a cooling rate of 0.01 K/s, the high-temperature (HT) reaction at 450 to 350 °C dominates corresponding to the precipitation of the S-phase (Al_2_CuMg)^8^. At cooling rate of 0.3 K/s, the medium-temperature (MT) reaction at 375 to 250 °C dominates corresponding to the η-phase (based on MgZn_2_)^8^. The low temperature (LT) reaction, dominant from about 250 to 150 °C corresponds to the previously unidentified precipitates that are the focus of this work. Following Milkereit, the area underneath the DSC peaks is the precipitation enthalpy or precipitation heat, which is directly proportional to the volume fraction of precipitation^7^. The total precipitation heat and precipitation heat values for each reaction are plotted as a function of cooling rate in Fig. 1(b). Details about the evaluation of the precipitation heat process can be found elsewhere^8^,^9^.

The overall precipitation heat decreases with increasing cooling rates, suggesting that quench-induced precipitation is gradually inhibited by increasing the cooling rate. The precipitation heat values for the HT S-phase gradually decrease with increasing cooling rate. This value approaches zero at cooling rates of 1–2 K/s, suggesting negligible amounts of S-phase precipitation occur above 1 K/s. However, the detected precipitation heat values for individual quench-induced precipitation reactions are not necessarily decreasing with increasing cooling rates^7^,^8^, as shown with the green MT reaction and blue LT reaction curves in Fig. 1(b). For example, the precipitation heat values for the MT precipitation of the η-phase increase over the cooling rate range from 0.01 to 0.1 K/s and then decrease at cooling rates above 0.3 K/s.

The same increasing trend can be found for the LT phase over the cooling rate range from 0.03 to 1 K/s. This behaviour can be ascribed in part to the suppression of the precipitation reactions at higher temperatures, resulting in an increased amount of solute becoming available for the LT phase precipitation. However, at a certain cooling rate, each diffusion-controlled precipitation reaction will still be suppressed by rapid cooling. It is therefore reasonable to assume that the LT phase will also be suppressed by rapid cooling at cooling rates above about 3–10 K/s (the limit of conventional DSC devices). Similar trends have been observed for 6xxx alloys at fast cooling rates^6^,^7^,^9^.

Since the precipitation heat for the η-phase rapidly approaches zero at cooling rates above 3 K/s, it is evident that LT phase precipitation increasingly dominates the total precipitation reaction at cooling rates of ≥3 K/s. The extrapolation of the precipitation heat at cooling rates above 3 K/s in Fig. 1(b) is based on a combined assessment of the DSC results as well as the trends in hardness, strength and observations of microstructural evolution as discussed below.

Figure 1(b) also shows that the ultimate tensile strength (UTS) tends to increase with increasing cooling rate. The UTS value in the T6 heat treatment condition reaches a maximum value of almost 650 MPa at the maximum cooling rate of 1000 K/s (25 °C water quench after solution annealing). This is an indication that all the solutionised solute atoms have been preserved in solid solution by the rapid quenching, leading to a maximum age hardening capability. Even if there is a small amount of quench induced precipitation in that cooling rate range, its small volume fraction will lead to a negligible effect on the overall strength.

The as-quenched strength curve shows similar trends, except that the maximum as-quenched strength of about 400 MPa that can be achieved occurs over a cooling rate range of 3–100 K/s. The as-quenched strength is then seen to decrease by about 50 MPa with increasing cooling rate from 100 to 1000 K/s. At cooling rates below 3 K/s, the as-quenched strength curve also exhibits a significantly decreasing trend with decreasing cooling rate. The yield strength (YS) and hardness values show similar trends, as illustrated in the supplementary materials.

**Figure 2:**
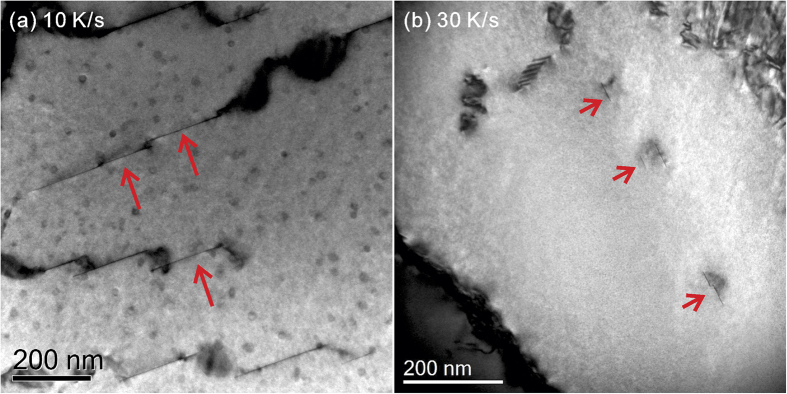
Bright field TEM micrographs showing the thin plate-shaped phase after cooling at (**a**) 10 K/s and (**b**) 30 K/s. The images are taken within subgrains along the [110]_α_ zone axis.

Figure 2(a,b) show bright field (BF) TEM images of samples cooled continuously to room temperature at cooling rates of 10 K/s and 30 K/s, respectively. Very few, if any, η-phase and S-phase precipitates can be detected in these cooling conditions. However, Fig. 2(a) shows that some platelet precipitates co-exist with some spherical precipitates in this cooling condition. These spherical precipitates are known to be Al_3_Zr dispersoids that formed during the homogenisation process^10^,^11^,^12^. The platelet precipitates are longer and present in larger amounts at the cooling rate of 10 K/s, than at the cooling rate of 30 K/s. It is therefore concluded that this platelet phase is a quench-induced phase since the growth of the platelet phase is gradually inhibited by increasing cooling rates. There are also some similar findings from DSC work on other alloys^7^,^13^,^14^. Compared to the S-phase and η -phase, the inhibition of growth of the platelet precipitates happens in a relatively fast cooling rate range (i.e. >3–10 K/s). Therefore it is concluded that the observed platelet phase corresponds to the low temperature precipitation reaction.

To the best knowledge of the authors, this platelet phase has never previously been reported in 7xxx series alloys, despite this system being widely used in aerospace applications. We have therefore named it the Y-phase. This work shows that an appreciable amount of this Y-phase precipitation only occurs under very particular cooling conditions i.e. during continuous cooling over a relatively fast cooling rate range from about 1 to 100 K/s and in a low temperature range of about 150–250 °C^8^. The amount of Y-phase increases with increasing cooling rate over the cooling rate range from about 0.03 K/s to about 3 K/s. It is possible that this Y-phase can precipitate in very slow cooling conditions, but the amount is very limited as the precipitation heat is very low. It is expected that the precipitation of this Y-phase will be totally suppressed by very rapid cooling above about 100–1000 K/s.

The as-quenched strength is seen to reach its maximum value over the cooling rate range of 3–100 K/s. This can be ascribed in part to the precipitation of the platelet phase, as the amount of the platelet phase is close to its maximum value in these cooling conditions. With further increases of cooling rate (>100 K/s), the amount of the platelet phase decreases, causing a corresponding decrease in the strength even though the solid solution hardening increment is expected to increase. According to Nie and Muddle, platelet precipitates on the 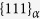 planes have the highest strengthening potential in aluminium alloys^15^. It is therefore not unreasonable to expect a strengthening increment from this platelet phase.

Based on the conventional bright field TEM images in Fig. 2, it is interesting to see that there is only one set of edge-on plates from the <110>_α_ direction (there are normally two sets of 111 plane precipitates visible in this direction) in any individual sub grain. This could be a strong indication of the nucleation route. The precipitate plates are heterogeneously distributed within subgrains. This may be due to preferential nucleated in subgrains rather than in recrystallised grains. However, the exact mechanism is as yet undetermined. The micrographs in Fig. 2 show that the Y-phase precipitates have a very low thickness and a very high aspect ratio (length/thickness ratio). This makes them clearly visible from an edge-on orientation, but almost invisible from other orientations.

**Figure 3:**
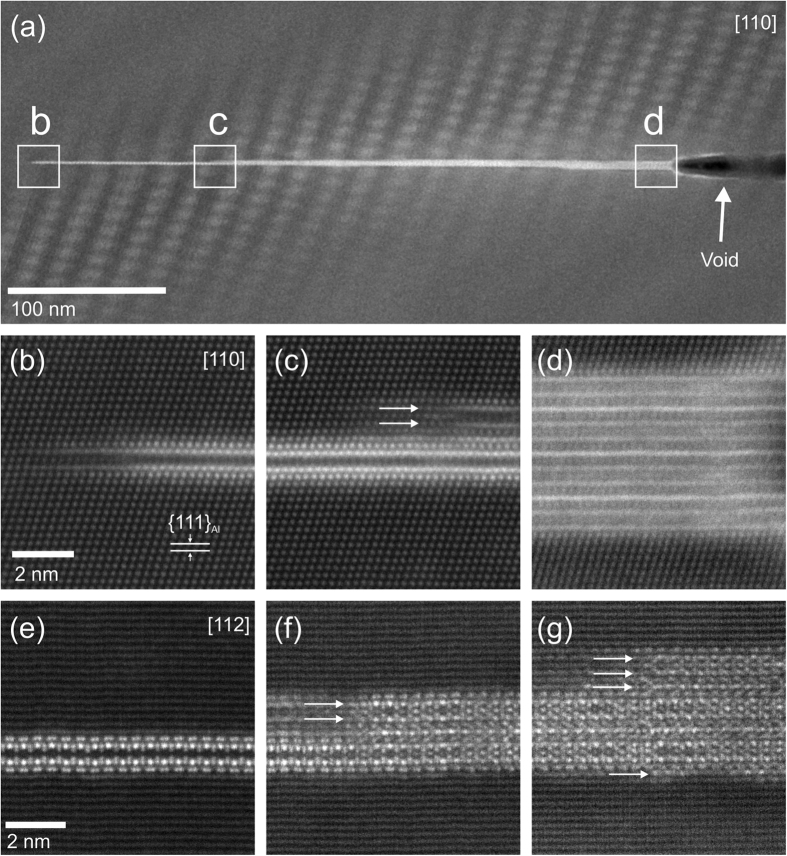
(**a–d**) HAADF-STEM images of a single platelet precipitate, viewed from the [110]_α_ direction, showing that the thickness varies along its length, with growth ledges indicated by arrows in (**c**). The regular pattern in the matrix on either side of the plate in (**a**) is an artifact caused by Moiré fringing between the lattice and scan frame. This plate appears to be nucleating from an attached void. Similar images in (**e–g**) from a second plate, but viewed from the [112]_α_ direction, show thickness and stacking order variations along the precipitate length.

Figure 3(a–d) show HAADF-STEM images of a single platelet precipitate with the electron beam parallel to the <110>_Al_ direction. The thickness of this platelet precipitate varies from a double-layer structure (as shown in Fig. 3(b)) to a multi-layer structure (as shown in Fig. 3(d)). Apparent growth ledges are indicated by the arrows in Fig. 3(c). Figure 3(e–g) show a second platelet with the electron beam parallel to the <112>_Al_ direction. The structure revealed in this direction indicates that this phase cannot be described by a regular 3D unit cell. The structure of the minimal thickness plate in Fig. 3(e), corresponding to a double bright layer with a dark center, is not replicated when the plate thickens (Fig. 3(f,g)). The apparent replication of various layers within the thicker sections may allow them to be described as long period stacking order (LPSO) structures, such as those common in magnesium rare earth alloys. However, such a description will require the systematic characterisation of a large number of precipitates. A recent study by Marioara *et al.* on a similar alloy system (AA7449) also observed structural arrangements in a new precipitate phase showing similarities to those of the T_1_ phase in Al-Cu-Li alloys^16^. While they did not observe any minimal thickness plates, possibly since they investigated over-aged samples, they tellingly suggested that “It may therefore be expected that in some conditions a plate isostructural to the T_1_ phase could also exist in the Al-Mg-Zn system”^16^.

**Figure 4:**
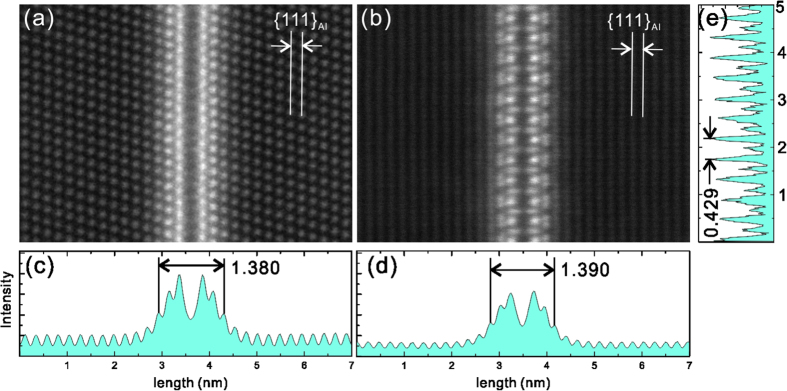
Atomic-resolution HAADF-STEM imaging of a single platelet precipitate viewed from (**a**) [110]_α_ and (**b**) [112]_α_ directions, with (**c**) and (**d**) showing respective plots of image intensity integrated vertically, and (**e**) showing the distribution of intensity along the length of the precipitate.

The projected atomic structure of the minimal thickness platelet bears a striking resemblance to plates of the T_1_ phase that are the principal strengthening phase in 2xxx series Al-Cu-Li alloys^15^,^17^,^18^,^19^,^20^,^21^,^22^,^23^. The images from two different observation directions in Fig. 4(a,b) strongly indicate that the Y-phase has a similar crystal structure and symmetry to the T_1_ phase. The T_1_ phase also shows long period stacking order in its thicker forms. On the basis of structural isomorphism with T_1_, the minimal thickness platelet has a hexagonal basis symmetry with repeat distances of a = 0.429 nm, c = 1.385 nm (T_1_ is in the P6/mmm space group with D_6h_ symmetry^23^), with the following orientation relationship with the matrix:


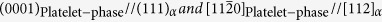


The measured parameters are based on HAADF-STEM images using the 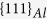 plane space (d_111_ = 0.2338 nm) as a reference. These are subtly different to the parameters for the T_1_ phase (a = 0.496 nm, c = 1.391 nm)^23^. It is noted that c is very close to 6 × d_111_ = 1.4028 nm, so that the platelet phase is slightly mismatched with the Al matrix, as shown in Fig. 4(a). This can be attributed to the atomic positions of the different solute species within the Y-phase. A previous study of the T_1_ phase showed that the T_1_ phase tends to precipitate by heterogeneous nucleation on dislocations in Al-Cu-Li alloys^24^. This may provide some clues for the nucleation mechanism of the platelet phase in alloy 7150, as the platelet precipitates are mainly found within subgrains. Based on Fig. 3(a), nucleation from voids is also a possibility.

**Figure 5:**
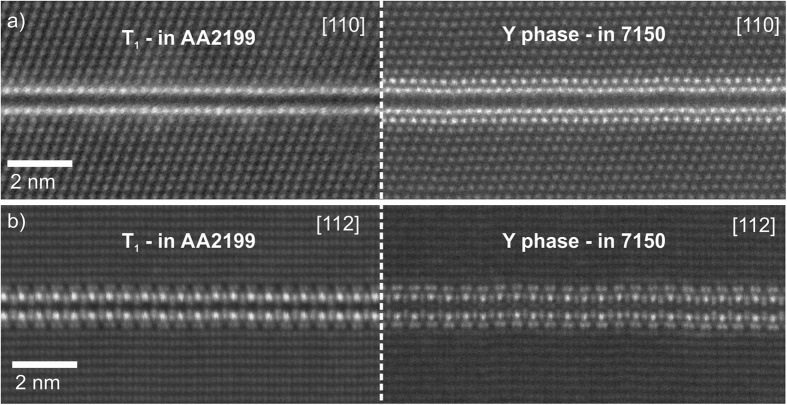
A direct comparison of the T_1_ phase in age hardened alloy AA2199 (left) with the Y-phase from alloy AA7150 (right), viewed from both (**a**) 110 and (**b**) 112 directions.

Figure 5 shows HAADF STEM micrographs comparing the T_1_ phase in an age hardened AA2199 alloy (left) with the Y-phase from alloy AA7150 (right), along both (a) 110 and (b) 112 directions. These micrographs were acquired on the same instrument using the same imaging and collection conditions (though with different, and undetermined, specimen thicknesses).

In the 112 direction there is strong similarity, with both phases showing a distinctive “zipper” appearance: bright columns separated by a dark band, with distinctive dumbbell structures on the surface and facing inwards. The appearance on 110 is similar, but the T_1_-phase lacks the extra surface layers (clearly present in 112)–in projection the Li content of these layers leads to an almost complete loss of contrast. Clearly the average atomic number of the bounding layer in the new phase is higher than that of bulk Al. This is consistent with the differing relative contrast in the bounding layer of the new phase, which is significantly brighter than the matrix.

While there is clear structural similarity between the Y-phase and the T_1_ phase^23^, alloy 7150 contains no Li so the chemistry of this phase must be substantially different. Due to electron channelling in crystalline specimens, quantitative chemical analysis on these length scales is not possible without a full solution of the atomic structure^25^ and precise knowledge of acquisition geometry^26^. However, both the EDX and EELS results from the plates give a qualitative indication of the chemical makeup of, and to some extent, the partitioning within the precipitate plates.

**Figure 6:**
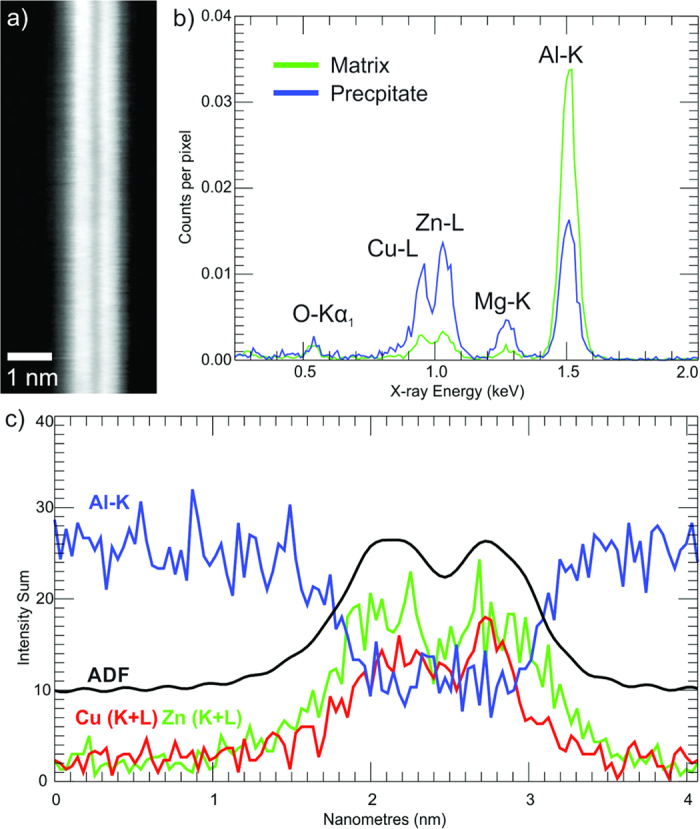
Results of EDX spectrum imaging (SI): (**a**) ADF image acquired simultaneously with EDX data; (**b**) Masked generated average spectra from precipitate and matrix; (**c**) Summed line trace across precipitate for ADF, Al-K, Cu (K + L) and Zn (K + L) signals.

The average EDX spectra, Fig. 6(b), taken from the precipitate shown in Fig. 6(a) confirm that the precipitates contain a significant proportion of Cu, Zn and a reduced amount of Al in comparison to the matrix. The precipitate spectra also indicate the inclusion of a small, yet significant, amount of Mg. The signal to noise ratios (SNR) of 2D maps from the EDX SI (Supplementary Fig. (4) are low but the summed line trace from the SI across the precipitate in Fig. 6(c), indicates that the high Z contrast layers peak in both Cu and Zn content, with Al present but at a low level across the plate.

**Figure 7:**
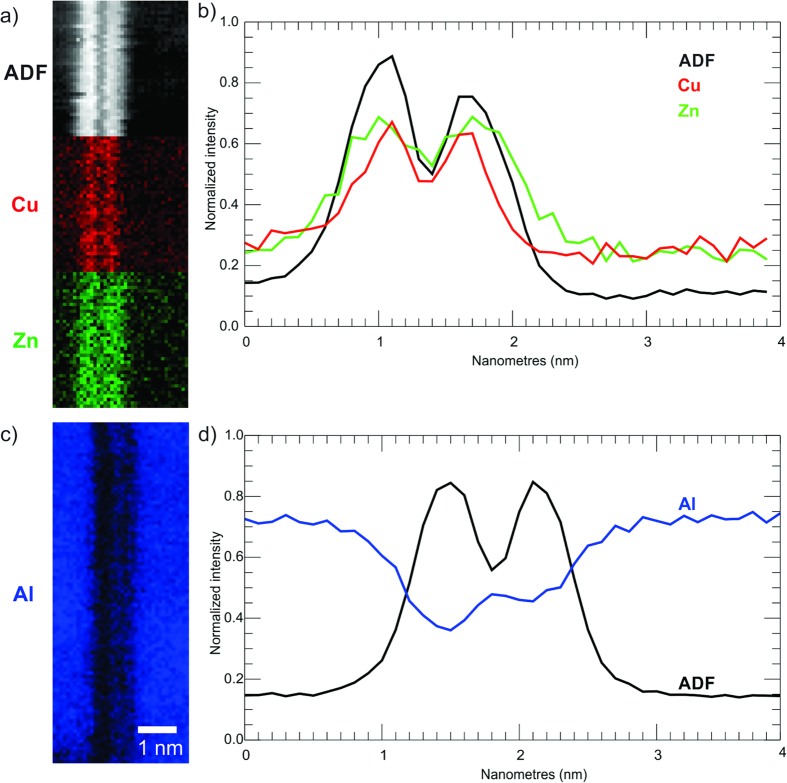
Results of two EELS spectrum images (SI), acquired from the same plate over different energy ranges: (**a**) ADF, Cu map and Zn map from first SI. (**b**) Summed line traces across precipitate for ADF, Cu map and Zn map; (**c**) Al map; (**d**) Summed line traces across Al map and ADF image (not shown).

The EELS SI results, shown in Fig. 7, are consistent with the EDX data and, being of significantly higher SNR, offer insight into the chemical structure of the precipitate. The maps, Fig. 7(a,c), and average line traces, Fig. 7(b,d), indicate that Cu has a narrower distribution within the precipitate, and is strongly peaked in the two bright layers. While Zn is also peaked in the same locations, its distribution is broader and extends into the bounding layers of the precipitate. Due to the large energy difference between Al-L and Cu-L/Zn-L edge onsets (73eV and 931 eV/1020 eV, respectively) it is not possible with the available spectrometer to map both in the same acquisition. Maps taken in a separate acquisition from the aluminium L edge from the same precipitate, Fig. 7(c) show a small increase in Al composition in the dark (low Z) centre layer of the precipitate plate. The asymmetry of this feature is probably due to a tilt misalignment of the crystal. In the very thin area used for the acquisition (7 nm via PACBED), exact on-axis conditions are hard to achieve. The same feature is weakly indicated in the EDX trace in Fig. 6(c). Despite the Mg-K edge being included in the energy range with the Cu and Zn K edges no peak was detected above the noise level. However this can be explained by looking at the relative scattering cross sections for the three edges. The inelastic scattering cross section for the Mg-K peak, calculated via the Hartree-Slater model in Digital Micrograph, is 7 times lower than for Cu-L and 5 times lower than Zn-L over a 50 eV energy window. As such, Mg has a much lower detectability than Cu or Zn over this energy range. While the Mg-L edge is contained in the spectra with the Al-K, there is also no edge present. The large amounts of Al, and the relatively low SNR of the Al-K map in Fig. 7(c), suggests that Mg is present in very small amounts, consistent with the EDX data. The T_1_ phase has also been shown to be able to incorporate small amounts of other elements, including Mg, during nucleation and growth^27^.

## Conclusions

This paper has reported the discovery of a new platelet phase that precipitates in alloy AA7150 during relatively fast continuous cooling in a relatively low temperature range of about 150–250 °C. These Y-phase precipitates with high aspect ratio grow on the aluminium {111}_*α*_ planes. The microscopy results show the Y-phase to be structurally very similar to the T_1_ phase found in Al-Cu-Li alloys, and to have a hexagonal symmetry (a = 0.429 nm, c = 1.385 nm). Tensile testing indicates that this Y-phase appears to contribute up to ~50 MPa to the as-quenched strength in the investigated continuous cooling conditions. It may also precipitate in other Cu-containing 7xxx alloys.

The results of the chemical analysis are consistent with the contrast observed in the HAADF images and the chemical differences to the T_1_ phase. The Y-phase is dominated by layers rich in Zn and Cu, the distinctive bright contrast in the 110 direction, with a centre plane dominated by Al and hence exhibiting darker contrast. The lack of Li in the structure compared to T_1_, results in the bounding planes being a mix of Al and Cu/Zn, with the EELS data suggesting these bounding planes are higher in Zn than Cu. The high average atomic number of these planes explains why they appear as brighter than the matrix in the 110 direction (as opposed to T_1_ where the presence of Li leads to an average atomic number similar to that of the matrix, with these layers practically invisible). While the EDX results show the presence of Mg in the plate, this is at a level too low to achieve any determination of its partitioning; indeed this was too low to even be detectable via EELS. A final solution of the precipitate phase structure is ongoing, but will require construction of prospective models, and matching with extensive image simulation and *ab initio* calculation.

## Additional Information

**How to cite this article**: Zhang, Y. *et al.* Precipitation of a new platelet phase during the quenching of an Al-Zn-Mg-Cu alloy. *Sci. Rep.* **6**, 23109; doi: 10.1038/srep23109 (2016).

## Supplementary Material

Supplementary Information
