# Supplementary material for: Precipitation of a new platelet phase during the quenching of an Al-Zn-Mg-Cu alloy
Source: Sci Rep. 2016 Mar 16;6:23109. doi: 10.1038/srep23109 (PMC4793252; doi:10.1038/srep23109)
Supplement: Supplementary Information [file srep23109-s1.pdf]

Precipitation of a new platelet phase during the quenching of an Al-Zn-Mg-Cu alloy

Yong Zhang [a,*], Matthew Weyland [a,b], Benjamin Milkereit [c,d], Michael Reich [c] Paul A Rometsch [a],

[a] Department of Materials Science and Engineering, Monash University, Clayton, VIC 3800, Australia

[b] Monash Centre for Electron Microscopy, Monash University, Clayton, VIC 3800, Australia

[c] Chair of Materials Science, Faculty of Marine Technology and Mechanical Engineering, University of Rostock, 18051 Rostock, Germany

[d] CALOR – Research Competence Center Calorimetry and Thermal Analysis Rostock, Faculty for Interdisciplinary Research, Department Light, Life and Matter, University of Rostock, 18051 Rostock, Germany

*Correspondence to tony.zhang@Monash.edu

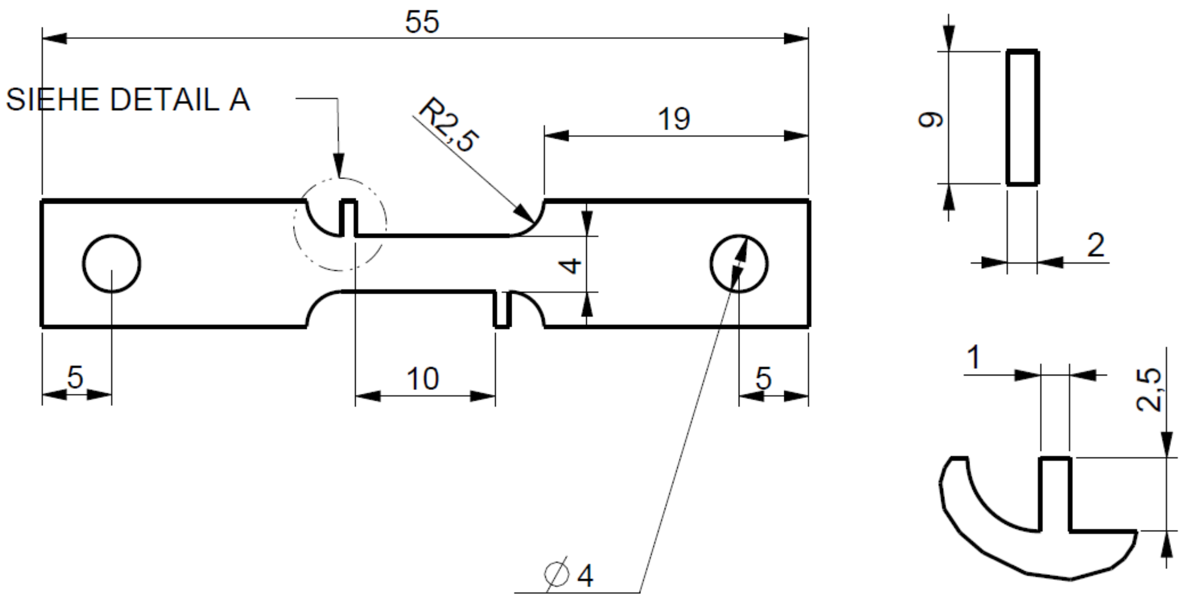

Figure S1: Geometry of dilatometer tensile test samples

**AA7150**	Solution Treatment: 460 °C 1 h + 480 °C 1 h								
Mass %	Si	Fe	Cu	Mn	Mg	Zn	Zr	Cr	Ti
	0.02	0.05	2.04	0.04	2.15	6.33	0.12	<0.01	0.01

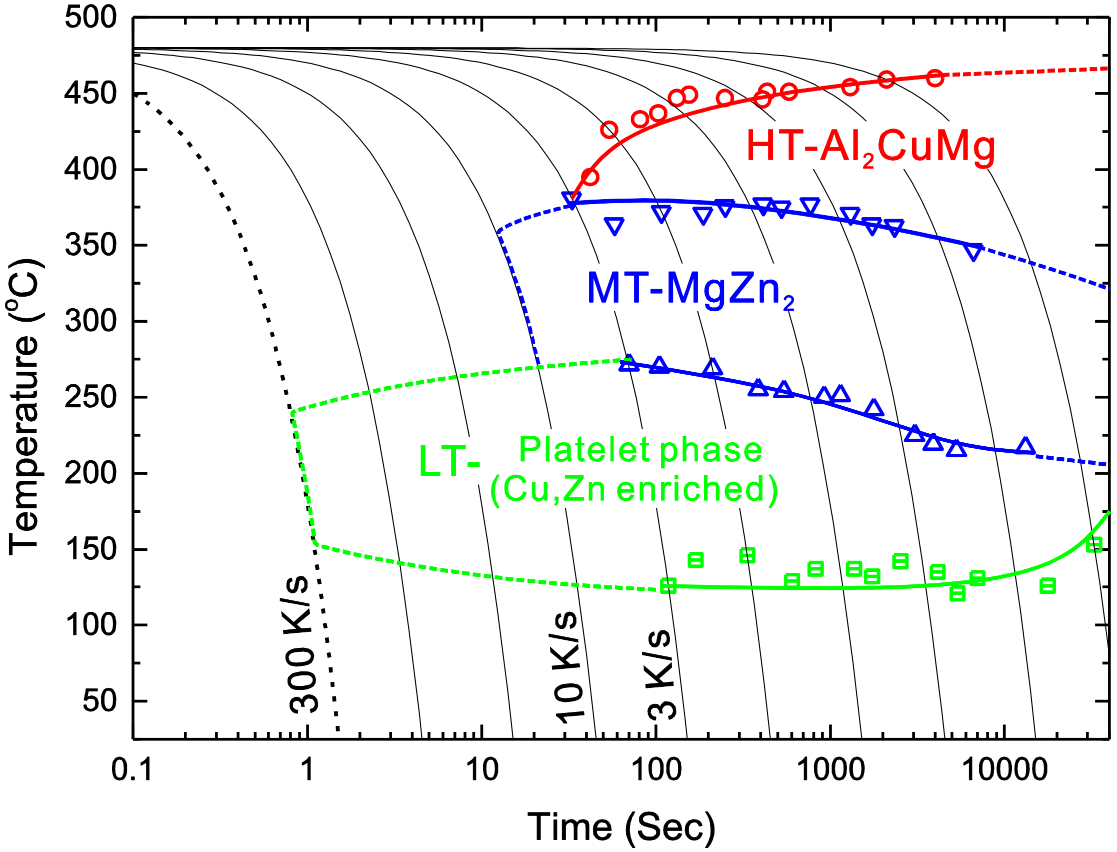

Figure S2: Developed continuous cooling precipitation (CCP) diagrams for the studied alloy AA7150 after solution treatment, showing linear cooling rates ranging from 300 K/s to 0.01 K/s. The dashed lines indicate regions where the precipitation reactions were extrapolated on the basis of combined evaluations of all the available DSC data, hardness curves and microstructural analysis [1].

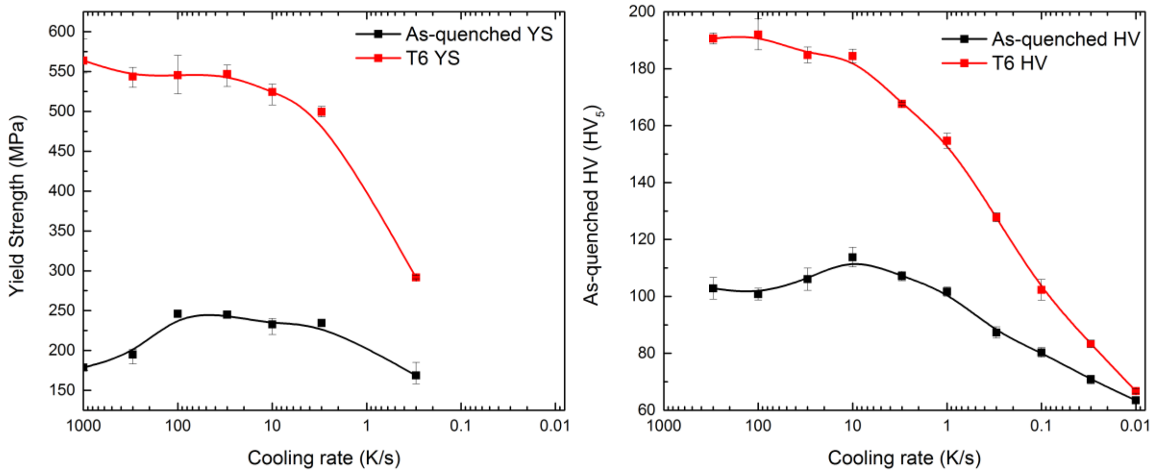

Figure S3: Yield strength and hardness curves at different cooling rates

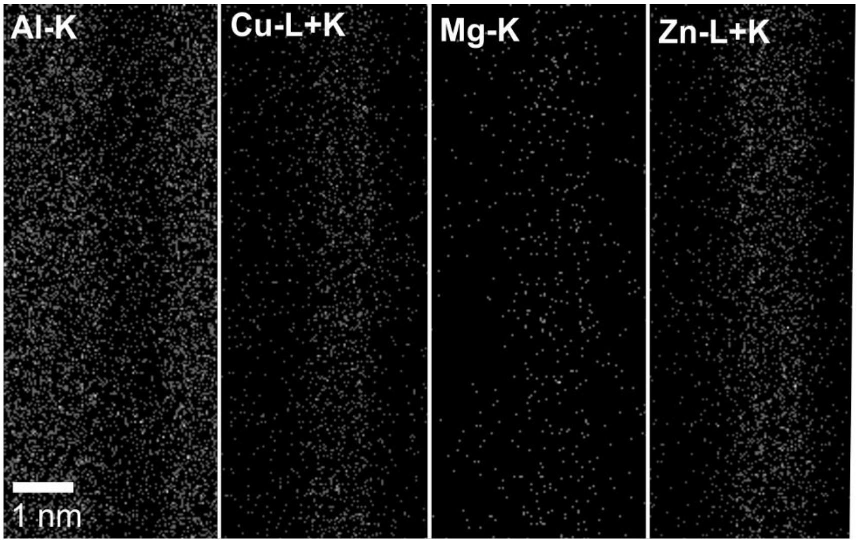

Figure S4: Corresponding 2D EDX maps which have been summed in the vertical direction to generate the line traces presented in Figure 6

References:

1 Zhang, Y., Milkereit, B., Kessler, O., Schick, C. & Rometsch, P. A. Development of continuous cooling precipitation diagrams for aluminium alloys AA7150 and AA7020. *J. Alloys Compd.* **584**, 581-589 (2014).
